# High dairy fat intake related to less central obesity: A male cohort study with 12 years’ follow-up

**DOI:** 10.3109/02813432.2012.757070

**Published:** 2013-06

**Authors:** Sara Holmberg, Anders Thelin

**Affiliations:** ^1^Research and Development Center, Kronoberg County Council, Växjö, Sweden; ^2^Department of Public Health and Caring Sciences, Family Medicine and Clinical Epidemiology Sections, Uppsala University, Uppsala, Sweden

**Keywords:** Abdominal obesity, butter, diet, general practice, metabolic syndrome, milk, saturated fat, Sweden

## Abstract

**Objective:**

To study associations between dairy fat intake and development of central obesity.

**Design:**

A prospective population-based cohort study with two surveys 12 years apart.

**Setting:**

Nine municipalities selected from different parts of Sweden representing the rural areas in the country.

**Subjects:**

1782 men (farmers and non-farmers) aged 40–60 years at baseline participated in a baseline survey (participation rate 76%) and 1589 men participated at the follow-up. 116 men with central obesity at baseline were excluded from the analyses.

**Main outcome measures:**

Central obesity at follow-up defined as waist hip ratio ≥ 1.

**Results:**

197 men (15%) developed central obesity during follow-up. A low intake of dairy fat at baseline (no butter *and* low fat milk *and* seldom/never whipping cream) was associated with a higher risk of developing central obesity (OR 1.53, 95% CI 1.05–2.24) and a high intake of dairy fat (butter as spread *and* high fat milk *and* whipping cream) was associated with a lower risk of central obesity (OR 0.52, 95% CI 0.33–0.83) as compared with medium intake (all other combinations of spread, milk, and cream) after adjustment for intake of fruit and vegetables, smoking, alcohol consumption, physical activity, age, education, and profession. The associations between dairy fat intake and central obesity were consistent across body mass index categories at baseline.

**Conclusion:**

A high intake of dairy fat was associated with a lower risk of central obesity and a low dairy fat intake was associated with a higher risk of central obesity.

Dietary advice is an important lifestyle intervention in primary care but what advice ought to be given?

- Recent research proposes that dairy products, rich in saturated fats, do not hold the negative metabolic consequences previously believed. Central obesity was studied in relation to dairy consumption among rural men.
- A high intake of dairy fat was associated with a lower risk of developing central obesity and a low intake of dairy fat was associated with a higher risk of central obesity.

## Introduction

Dietary advice is a lifestyle intervention applied for primary and secondary prevention in primary care. But what diets and foods are to be recommended is partly uncertain and subjected to scientific debate [1,2]. Milk and dairy products are traditional components of the Scandinavian kitchen but especially high-fat dairy has for long been disapproved of in health recommendations due to the high content of saturated fats believed to generate heart disease. However, a large meta-analysis of prospective studies published in 2010 showed no evidence for any association between dietary saturated fat and the risk of cardiovascular disease [3]. Other reviews have come to similar conclusions concerning mortality, heart disease, stroke, and diabetes [4,5]. Another recent meta-analysis reported a modest inverse association for milk intake and risk of overall cardiovascular disease but no association with coronary heart disease, stroke, or total mortality [6].

Central or abdominal obesity indicates insulin resistance and is part of the metabolic syndrome and well known to increase the risk of diabetes but it is also associated with heart disease [7], various cancers [8,9], dementia [10], and preterm death [11]. Prevention of central obesity is therefore crucial with the potential of limiting the risk for various diseases.

Several studies have shown inverse relationships between dairy consumption and metabolic risk factors and central obesity [12–14] while others showed the opposite or no association [15–18]. Whole milk and dairy products are complex foods with many important nutrients and potential anti-nutrients and the precise mechanisms explaining the effects on health have not been clarified [4,19–21]. However, from a clinical perspective better understanding of the health effects related to intake of various foodstuffs is of greater importance than the exact understanding of molecular mechanisms.

We have followed a cohort of rural men over 12 years [22]. In a previous study we found that daily intake of fruit and vegetables in combination with a high dairy fat intake was associated with a lower risk of coronary heart disease [23]. Our present aim was to study how dairy fat intake impacts on the risk of developing central obesity in this middle-aged male cohort.

## Material and methods

### Study population

A research project on health and work focusing on health-promoting factors among farmers was designed in 1989. A study cohort with farmers and rural referent was established. All male farmers born between 1930 and 1949 living in nine selected rural municipalities in Sweden were identified from the Swedish National Farm Register. Occupational activity in farming was thoroughly checked. For each farmer a rural referent, matched by age, sex, and residential area, was identified in the National Population Register. The referents were to be occupationally active, in work other than farming, according to the most recent census.

Altogether 2350 men (1220 farmers and 1130 non-farmers) met the criteria and were included in the cohort. They were invited to an extensive health survey in 1990–91 including questionnaires, interviews, physical examinations, and laboratory test. The participation rate in this baseline survey was 75.8% [24].

The entire cohort was invited to a follow-up survey in 2002–03. The participation rate at this examination was 67.6%. In total 1405 men participated in both surveys, that is 59.8% of the initial cohort and 63.6% of those still alive at the time for survey 2.

The health examinations were performed by specially trained teams of physicians and nurses traveling out to the various areas. The surveys were done solely for research purposes and hence were not part of any ordinary or local health program.

The study was approved by the Research Ethics Committee at the Karolinska Institute in Stockholm, Sweden and by the Regional Ethics Board, Uppsala, Sweden. All men who participated in the health surveys gave their informed consent.

### Outcome

Central obesity was defined as waist hip ratio ≥ 1. Waist and hip measurements were taken at both surveys with a tape measure at the level of the umbilicus and at the widest part of the hips with the participants dressed in light wear.

### Dairy fat intake

Specific food choices were assessed by a 15-item questionnaire answered at the two surveys. Dairy fat intake was assessed by combining three questions relevant to Swedish eating habits, namely usual spread on sandwiches (butter, low fat margarine, or no fat), type of milk normally consumed (non- homogenized farm milk, full fat milk with 3.0% fat, semi-skimmed with 1.5% fat, or skimmed milk with 0.5% fat) and intake of whipping cream, also in sauces (daily, sometimes during the week, or seldom/never). Low consumption of dairy fat was defined as no butter, low fat milk (1.5% fat or less), and seldom or never intake of cream. High consumption of dairy fat was defined as butter as spread, full fat milk, and intake of whipping cream daily or several times a week. All other combinations of spread, milk, and cream consumption were defined as medium dairy fat intake.

### Potential confounders

In the food questionnaire one item assessed intake of fruit and berries (nearly every day, several times a week, once a week, or seldom/never) and one item assessed intake of vegetables, legumes or root vegetables, except potatoes, with the same alternatives as for fruit and berries. These items were combined into one variable for fruit and vegetables dichotomized as daily versus less than daily intake.

Weight and height were measured with standard procedures and body mass index (BMI) was calculated as weight in kilograms divided by height in meters squared. Tobacco use, alcohol consumption, and physical activity were assessed in detail in structured interviews. Smoking habits were for these analyses dichotomized as current daily smoking versus no smoking. Alcohol consumption was assessed as frequency of use and amount of alcoholic beverages consumed on each occasion. Average alcohol intake was then computed as grams of pure alcohol consumed per week. Physical activity during leisure time was assessed on a four-grade scale from sedentary to vigorously active. Educational level was classified as mandatory, vocational school, secondary school, college, or university according to self-report in questionnaire.

### Statistical analyses

The internal non-response rate was low for all included variables. Data analyses were performed using SPSS^^®^^ version 14.0. A significance level of 0.05 was considered to indicate statistical significance and all tests were two-tailed. Analyses of association were performed with logistic regression in multiple analyses adjusting for confounder variables. The results are presented as odds ratios (OR) with 95% confidence intervals (95% CI). Confounder variables were categorized and eliminated from the model in a backward selection of non-significant variables requiring a p-value below 0.10 for the variable to be kept in the model.

## Results

Baseline descriptive statistics are presented in Table I. The majority were overweight or obese according to BMI and 6.5% were centrally obese (waist hip ratio ≥ 1). One-fifth (20.4%) were categorized as having a low dairy fat intake at baseline and almost one-fourth (23.7%) had a high dairy fat intake. One-third reported consuming fruit *and* vegetables daily.

**Table I.** Descriptive statistics of participants at baseline in 1990–91 (n = 1782).

	n	%	mean	SD	min	max
Age (years)	1,782		50.3	6.0	39	62
Farmer	1,013	56.8				
Body mass index (BMI)	1,782		26.4	3.2	18.3	44.4
Normal weight (BMI < 25)	634	35.6				
Overweight (BMI 25–29.9)	897	50.3				
Obesity (BMI ≥ 30)	251	14.1				
Waist hip ratio (W/H)	1,771		0.91	0.05	0.71	1.11
Central obesity (W/H ≥ 1.0)	116	6.5				
Smoking, daily	414	23.3				
Alcohol consumption (grams/week)	1,777		24.9	29.3	0	227
≥ 60 grams/week	187	10.5				
Physical activity, leisure time	1,768					
Sedentary	510	28.8				
Low	997	56.4				
Moderate or vigorous	261	14.8				
Educational level	1,737					
Mandatory school	722	41.6				
Vocational school	551	31.7				
Secondary school	185	10.7				
College	123	7.1				
University	156	9.0				
Food choices						
Spreads						
Low fat margarine or nothing	915	51.7				
Butter	854	48.3				
Milk						
Low fat	813	48.9				
High fat	849	51.1				
Whipping cream						
Seldom/never	725	41.3				
Daily or several times a week	1,013	58.7				
Low fat (no butter+ low fat milk+ no cream)	356	20.4				
High fat (butter+ fat milk+ cream)	415	23.7				
Fruit *and* vegetables, daily	592	33.5				
Fruit, daily	936	52.8				
Vegetables, daily	917	51.8				

Among those without central obesity at baseline (1,322 men), 197 men (14.9%) developed central obesity during follow-up. The use of butter as spread, intake of high-fat milk, and intake of cream daily or several times a week at baseline were in crude analyses related to lower rates of central obesity at follow-up (Table II). High alcohol consumption was associated with central obesity. Higher physical activity, higher education, and being a farmer were associated with a lower rate of central obesity.

**Table II.** Central obesity (waist hip ratio ≥ 1) at follow-up (2002–03) with regard to reported food choices and other lifestyle variables at baseline among men without central obesity (waist hip ratio < 1) at baseline (n = 1,322).

	n	Cases of central obesity at follow up	%	OR^1^	95% CI
Spreads					
Low fat margarine or nothing	676	117	17.3	1	
Butter	636	75	11.8	0.64	0.47–0.87
Milk					
Low fat	609	106	17.4	1	
High fat	629	75	11.9	0.64	0.47–0.88
Whipping cream					
Seldom/never	523	92	17.6	1	
Daily or several times a week	787	101	12.8	0.69	0.51–0.94
Fruit and vegetables					
Less than daily	868	126	14.5	1	
Daily	445	68	15.3	1.06	0.77–1.46
Smoking					
Non-smokers	1,053	148	14.1	1	
Smokers	267	48	18.0	1.34	0.94–1.92
Alcohol consumption					
< 60 grams/week	1,194	168	14.1	1	
≥ 60 grams/week	124	28	22.6	1.78	1.13–2.80
Physical activity, leisure time					
Sedentary	392	69	17.6	1	
Low	712	102	14.3	0.78	0.56–1.09
Moderate or vigorous	207	21	10.1	0.53	0.31–0.89
Education					
Only mandatory school	507	90	17.8	1	
More than mandatory	788	100	12.7	0.67	0.49–0.92
Profession					
Non-farmer	544	97	17.8	1	
Farmer	778	100	12.9	0.68	0.50–0.92

^1^Crude odds ratio with 95% confidence intervals.

Among those without central obesity at baseline central obesity was developed by 19.9% of the men reporting low dairy fat intake at baseline and by 8.6% of those reporting a high dairy fat intake. In the intermediary group 15.1% developed central obesity. A low dairy fat intake was associated with higher risk of developing central obesity also after adjustment for possible confounders (1.53; 1.05–2.24) whereas a high dairy fat intake was associated with a lower risk (0.52; 0.33–0.83) (Table III). No significant interactions between included variables were identified.

**Table III.** Risk of central obesity (waist hip ratio ≥ 1) at follow-up according to dairy fat intake at baseline. Only men with waist hip ratio < 1 at baseline were included.

Dairy fat intake	Crude (n = 1,303)		Model 1^1^ (n = 1,285)		Model 2^2^ (n = 1,261)	
	OR^3^	95% CI	OR^3^	95% CI	OR^3^	95% CI
Low (no butter and low fat milk and seldom/never whipping cream)	1.40	0.97–2.03	1.45	0.99–2.11	1.53	1.05–2.24
Medium (all other combinations of spread, milk, and whipping cream)	1		1		1	
High (butter and high fat milk and whipping cream daily or several times a week)	0.53	0.34–0.83	0.50	0.31–0.80	0.52	0.33–0.83

^1^Adjusted for fruit and vegetables daily, smoking, alcohol consumption, and physical activity.

^2^Adjusted as above plus age, education, and profession.

^3^Odds ratio with 95% confidence intervals.

The associations between levels of dairy fat intake and central obesity were consistent across BMI categories at baseline. Smoking rate decreased from 23.3% at baseline to 14.3% at follow-up. However, there were no significant differences in central obesity according to change in smoking habits.

The number reporting a low dairy fat intake increased from 20.4% at baseline to 26.1% at the second survey and the number reporting a high dairy fat intake decreased from 23.7% at baseline to 17.1% at the follow-up. The number reporting daily intake of fruit *and* vegetables increased somewhat. However, few individuals made large dietary changes. Only two men shifted category from low dairy fat consumption to high dairy fat consumption and 20 individuals shifted from high dairy fat to low dairy fat consumption.

## Discussion

We found that a low intake of dairy fat was associated with a higher risk of developing central obesity and that a high intake of dairy fat was associated with a lower risk of central obesity among men without central obesity at baseline. The majority of the participants were overweight or obese as defined by BMI at baseline. However, the associations between dairy fat intake and central obesity were consistent across BMI categories at baseline.

The main strength of the present study is the prospective population-based design with a large study population. We consider the participation rate high in view of the considerable efforts required by participants in attending two extensive health surveys 12 years apart. The study population is fairly homogeneous, representing middle-aged rural occupationally active and relatively healthy males [22]. Another strength is that anthropometrics were measured in standardized ways and not self- reported. A large number of potential confounders were accessible and included in the analyses and there was generally a low internal non-response for included variables.

The main limitation of our study is the observational design. Randomized controlled trials are preferable but very difficult to accomplish due to the large challenges with dietary long-term studies in real-life environments. Second best is well- controlled prospective epidemiological studies. Another limitation may be the rough classification of foods in the questionnaire. The questionnaire was developed around the year 1990 according to the dietary conceptions discussed in the medical literature at that time. Accumulated knowledge over the follow-up period warrants perhaps other questions to be included. However, this is the situation for long-term studies, which have to deal with this dilemma. Milk, butter, and cream were considered but this does not cover all dairy products. Cheese and yoghurt for example were not included/not asked about, nor the vast list of processed dairy products available in the supermarkets of today.

Although several confounding variables were included in our analyses there may still be uncontrolled confounding. However, in view of the small effect on the results by the variables included it is unlikely that including further potential confounders would change the result substantially.

Our observational results are in concordance with other studies with observed benefits of dairy consumption [4,13]. A recent study shows that high plasma-levels of trans-fatty acids specific to whole- fat dairy consumption is associated with lower insulin resistance and lower incidence of diabetes [25]. Others have shown that low-fat dairy especially is related to a lower risk of diabetes [26]. Components other than fat content such as calcium and milk proteins in dairy products have been discussed [27]. Both positive and negative effects are possible from milk and dairy consumption and individual sensitivity might be pertinent. However, from a clinical perspective of dietary counseling in health care the net health effects evaluated in clinical studies are of greatest importance.
